# Effects of a Patient-Provider, Collaborative, Medication-Planning Tool: A Randomized, Controlled Trial

**DOI:** 10.1155/2016/2129838

**Published:** 2016-09-06

**Authors:** James F. Graumlich, Huaping Wang, Anna Madison, Michael S. Wolf, Darren Kaiser, Kumud Dahal, Daniel G. Morrow

**Affiliations:** ^1^Department of Medicine, University of Illinois College of Medicine at Peoria, 530 Northeast Glen Oak Avenue, Peoria, IL 61637, USA; ^2^Department of Medicine, Division of Research Services, University of Illinois College of Medicine at Peoria, One Illini Drive, Peoria, IL 61605, USA; ^3^Department of Psychology, University of Illinois at Urbana-Champaign, 603 E. Daniel, Champaign, IL 61820, USA; ^4^Division of General Internal Medicine and Geriatrics, Department of Medicine, Northwestern University Feinberg School of Medicine, 750 North Lake Shore Drive, 10th Floor, Chicago, IL 60611, USA; ^5^Northwestern Medical Faculty Foundation, 675 North Saint Clair Street, Chicago, IL 60611, USA; ^6^Department of Medicine, University of Illinois College of Medicine at Peoria, One Illini Drive, Peoria, IL 61605, USA; ^7^Department of Educational Psychology, University of Illinois at Urbana-Champaign, Education Building, 1310 South 6th Street, Champaign, IL 61820, USA

## Abstract

Among patients with various levels of health literacy, the effects of collaborative, patient-provider, medication-planning tools on outcomes relevant to self-management are uncertain.* Objective*. Among adult patients with type II diabetes mellitus, we tested the effectiveness of a medication-planning tool (Medtable*™*) implemented via an electronic medical record to improve patients' medication knowledge, adherence, and glycemic control compared to usual care.* Design*. A multicenter, randomized controlled trial in outpatient primary care clinics. 674 patients received either the Medtable tool or usual care and were followed up for up to 12 months.* Results*. Patients who received Medtable had greater knowledge about indications for medications in their regimens and were more satisfied with the information about their medications. Patients' knowledge of drug indication improved with Medtable regardless of their literacy status. However, Medtable did not improve patients' demonstrated medication use, regimen adherence, or glycemic control (HbA1c).* Conclusion*. The Medtable tool supported provider/patient collaboration related to medication use, as reflected in patient satisfaction with communication, but had limited impact on patient medication knowledge, adherence, and HbA1c outcomes. This trial is registered with ClinicalTrials.gov NCT01296633.

## 1. Introduction

Medication is central to treating and managing type II diabetes mellitus, a prevalent age-related chronic illness [1]. Effective treatment is often undermined by nonadherence, with as many as half of patients not taking medications as prescribed [2, 3].

Nonadherence is traced to many causes but often involves a gap between the cognitive demands of adherence and inadequate cognitive resources that patients bring to the task, a problem that is compounded by limited healthcare system support [4]. For example, to manage complex medication regimens, patients with type II diabetes must create plans for taking multiple medications that meet constraints such as avoiding medication interactions and timing with respect to meals or other daily events. Planning requires cognitive resources related to health literacy [5–7], such as processing capacity (e.g., working memory) and health knowledge [8]. Nonadherence increases with regimen complexity [9], in part because of inadequate planning [5]. Older adults are especially likely to demonstrate nonadherence because they tend to have more complex medication regimens, yet experience declines in literacy and cognitive resources needed for self-care [8].

Healthcare system support for adherence is often inadequate [2]. For example, patient-provider collaboration is crucial for adherence [10, 11]. Education by providers can increase patient knowledge and self-care skills, and simplifying regimens and coordinating treatment across providers reduce adherence demands on patient cognitive resources. However, effective collaboration requires patients and providers to work together to ensure information is mutually understood [12], and providers do not always collaborate with patients effectively. While providers do most of the talking during consultations [13], they may skip key information [14], use non-patient-centered language [15], or fail to check patients' comprehension of the information that they present [16]. Medication review with patients is sporadic and fragmented [17] and reconciliation, the process of ensuring accurate, complete, and current patient medication lists, is often inadequate [2]. As a consequence, patients leave consultations with deficits in memory for important information and inadequate plans for self-care [18].

Adults with lower health literacy and cognitive resources are especially vulnerable to inadequate collaboration with providers. Patients with diabetes and lower health literacy report worse communication with providers [19] and have worse outcomes than do patients with adequate literacy [20, 21]. Adults with complex regimens and multiple self-care needs are candidates for system support because they are less likely to develop shared adherence plans with their providers, leading to nonadherence [2, 22].

Inadequate collaboration reflects barriers such as limited contact time and lack of support for consistent use of patient-centered communication strategies [2]. A promising approach is multimedia support for patient/provider collaboration. Patient memory for self-care information is improved when information is provided visually (text and graphics) as well as verbally during clinic visits, especially when the presented information is consistent, standardized, and embedded in structured processes that activate patients [13, 23]. Well-designed information technology can support multimedia approaches to patient-centered communication [4], but this potential has yet to be realized. For example, comprehensive medication lists printed on cards are recommended for medication review and reconciliation with patients, but studies evaluating such cards in pharmacy [24], hospital discharge [25], and specialized clinic [26] environments produced inconsistent evidence. This finding may reflect the fact that the cards were not specifically designed to support patient-provider collaboration nor were they linked with health information technology, thus not integrated with clinical practice.

We developed a patient education tool called the Medtable that is integrated with the electronic medical record (EMR) in primary care clinics [27]. The purpose of the Medtable is to improve patient self-management via patient-provider collaboration. Guided by distributed cognition theory, which suggests that cognitive activity can be effectively distributed across individuals (such as nurses and patients) and external artifacts (tools such as computers or paper) to support collaboration [28], the Medtable was designed to accomplish three goals: (1) to promote patient knowledge by clearly conveying accurate and relevant medication information; (2) to support collaborative planning wherein a patient, guided by a nurse, could efficiently organize medications tailored to his or her daily schedule to support use; (3) to embed the tool into clinical practice by integrating it with EMR systems so that it is easily updated, reliable, and shareable with providers.

Our use of EMR-integrated technology to support collaborative planning for medication use is unique in the literature on medication adherence among patients with diabetes. Few previous studies focus on patient/provider consultation (for review, see [29]). For example, one study assesses the use of paper-based tools to support patient/provider planning about medication taking [30]. Other studies evaluate problem solving protocols to address barriers to adherence during face-to-face [30–32] or telephone-based [33] communication. These studies do not involve the use of EMR-integrated tools designed to support specific cognitive processes underlying patient/provider collaboration and learning.

This EMR-enabled Medtable strategy was evaluated to determine its impact on medication use and health outcomes among patients with type II diabetes mellitus. The intervention involved nurses using the tool to support patients' medication planning. We hypothesized that, compared to usual care, patients randomized to this intervention will have greater medication knowledge, adherence, and better outcomes (as measured by glycosylated hemoglobin HbA1c levels), as well as being more satisfied with provider communication about medications. A secondary hypothesis was that intervention benefits would be greater for patients with lower health literacy than for those with adequate literacy, because the intervention was designed to address literacy-related barriers.

## 2. Methods

The study design was a two-arm, patient-randomized, controlled trial. Details about the trial design have been published [27]. The trial settings were outpatient primary care clinics in Chicago and Peoria, Illinois. All the research sites used the same electronic medical record and version (Epic, Verona, Wisconsin). The institutional review boards of Northwestern University and the University of Illinois approved the research. A group of experts comprised the Data Safety and Monitoring Board that monitored the trial and reviewed protocol changes.

### 2.1. Inclusion and Exclusion Criteria

Criteria for enrollment were (a) age 40 years and older; (b) native speaker of English; (c) no physical or cognitive impairments that could limit participation (e.g., stroke in the last 3 years, current cancer treatment involving radiation or chemotherapy); (d) score of 4 or higher on the short screen for dementia [34]; (e) no severe visual impairment (less than 20/50 corrected vision) or auditory impairment that would limit participation; (f) diagnosis of type II diabetes mellitus; (g) taking at least 5 prescribed medications; and (h) glycosylated hemoglobin (HbA1c) level of 7.0% or higher. The inclusion/exclusion criteria related to language proficiency and physical, sensory, and cognitive impairment were designed to minimize factors that might reduce response to the intervention and confound interpretation of the findings.

### 2.2. Participant Recruitment

Recruitment occurred from ambulatory care general internal medicine clinics in Chicago and Peoria, Illinois, which served as the performance sites for this study. Primary care physicians gave permission to screen their patient panels for potential participants who had the appropriate age, HbA1c, and number of medications. Then, potential participants received a letter via mail that described the research and said the patient would be contacted via telephone. Shortly before a scheduled clinic visit with the primary care clinician, clinical research coordinators contacted potential participants via phone to provide a questionnaire for inclusion/exclusion criteria, determine eligibility, and initiate the informed consent process. Participants who provided informed consent via telephone were scheduled for the baseline research visit that coincided with the next clinic visit with the primary care clinician. Participants completed the informed consent process at the baseline research visit and then immediately received the randomized intervention. Because of slow recruitment, the Data Safety and Monitoring Board authorized a change in the inclusion criteria to enroll participants with HbA1c of 6 or more.

### 2.3. Intervention: Medtable

**Figure 1:**
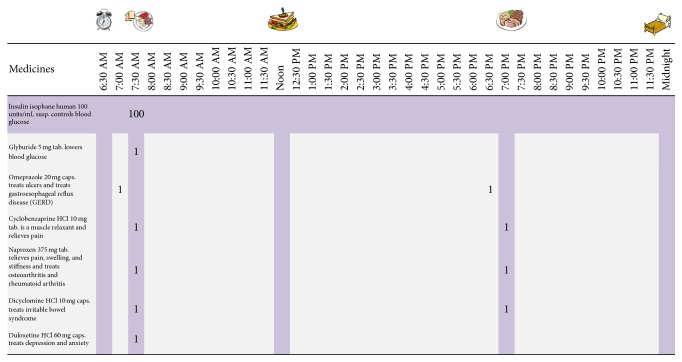
Example of Medtable. The patient and provider collaborate to choose times for each medication in the regimen. Modified and reprinted from [27] with permission from Elsevier.

Patients who were allocated to the experimental condition received the Medtable-based intervention (see Figure 1). A complete description of the Medtable has been published [27]. In summary, the Medtable is a structured tool that was implemented within the electronic medical record (EMR) at the outpatient clinics. The goal of the Medtable was to organize collaborative, patient/provider interactions for medication review, reconciliation, and education. Features of the Medtable included searchable libraries of medication administration instructions in direct, actionable language, timelines that support text, and familiar icons that represent key daily events. Implementation of the tool occurred during routine clinic visits, and this occurred in three stages. During the setup stage and prior to the patient visit, the nurse loaded the patient medication list from the EMR into the Medtable. At this stage, the nurse used the Medtable to customize the technical language from the EMR to provide language appropriate for patients with low health literacy. The second stage occurred with the patient during the clinic visit. The patient reviewed the EMR-based medication list, and then the nurse and patient collaboratively reconciled the list. The nurse added or deleted information in the EMR in response to the reconciliation stage. The goal of the second stage was an accurate and current medication list.

In the final stage, the patient and nurse jointly created a medication schedule while using the Medtable tool. Patients described their daily routine so the nurse could set up the tool around the routine. The Medtable displayed icons and highlighted columns to which the patient and nurse could refer while developing the schedule. The nurse and patient scheduled each medication by clicking on the cell in the table corresponding to the medicine (row) and the time slot (column). In this way, the tool scaffolded collaborative planning for taking the patient's medications. The nurses also discussed how to take each medication with the patients and used teach-back techniques to ensure patient comprehension. At the end of the third stage, the patient received a paper copy of the Medtable-based summary of their daily medication schedule to take home.

The intervention nurses were trained to use the Medtable as part of patient-centered care. Education for intervention nurses involved several components. Nurses received a multimedia manual with project overview, rationale for the intervention, overview of the Medtable tool and how it is used, and specific information about Medtable procedures. The education emphasized teach-back and teach-to-goal strategies to ensure patients understand how to take their medicines. While training, nurses interacted with the Medtable as patients as well as providers to optimize understanding from multiple perspectives. Nurses participated in simulated patient encounters to set up the tool and work with patients to develop schedules for medication regimens of varying complexity. To ensure fidelity of the intervention to the research protocol, the research personnel observed intervention nurses while working with several actual patients at both research sites. Feedback was provided to the nurses to reinforce initial training and ensure consistent delivery of the intervention across sites [27].

### 2.4. Usual Care

Patients allocated to usual care received medication counseling and communication from clinic nurses according to the standard of care at the research sites. Usual care included reconciliation of the patient's list of medications. The medication instructions on the list were comparable to the text commonly found on prescription labels. Usual care recipients and their providers did not receive prompts to organize the medication list around the patient's daily activities.

### 2.5. Measurement of Knowledge

The primary prespecified outcomes were verbal and demonstrated knowledge of the medication regimen [5, 35]. Research personnel assessed medication knowledge at baseline, immediately following the research intervention, and then 3 and 6 months later. Patients received a reminder to bring current prescription medication bottles or containers to each study visit. Clinical trial coordinators recorded all medications and dose directions from the label. To assess verbal knowledge of directions for use of injectable medications like insulin, clinical trial coordinators recorded the patient's responses to two questions: “On a usual day, how many times a day do you take this medicine?” and “How many units of this medicine do you usually take each time?” We scored correct verbal knowledge per injectable medication if the patient answered both questions correctly when compared to directions on the label. To assess verbal medication knowledge of directions for use of noninjectable, prescribed medications, clinical trial coordinators recorded the patient's responses to three questions for each medication: “On a usual day, how many times a day do you take this medicine?” and “How many pills of this medicine do you take each time?” and “How many pills of this medicine do you take each day in total?” For noninjectable medications, we scored correct verbal knowledge per medication if the patient answered all three questions correctly when compared to directions on the label. For purposes of analysis, we calculated combined verbal knowledge of the regimen for all questions: the number of medications scored as correct verbal knowledge divided by the total number of medications in the regimen.

Another verbal medication knowledge item was indication for each medicine in the patient's regimen. Older and less educated adults are less likely to know the purpose of their medications [36]. Clinical trial coordinators recorded the verbatim response to the following question: “What is the medicine for?” For purposes of analysis, we calculated combined knowledge of the indication for drugs in the regimen: the number of medications scored as correct indication knowledge divided by the total number of medications in the regimen.

We also assessed demonstrated medication knowledge for each noninjectable drug in the regimen. Clinical trial coordinators asked patients to show how they would take each of their medicines by placing beads (representing pills) into a pillbox that was partitioned into 24 slots, each slot representing an hour of the day [5]. We scored correct demonstrated knowledge per medication if the patient correctly demonstrated all 4 of the following: number of pills per dose, number of doses per day, number of pills each day in total, and amount of time (spacing) between doses. Combined demonstration knowledge of the regimen was the number of medications scored as correct demonstrated knowledge divided by the total number of medications in the regimen.

Scoring of the primary outcome was a blinded process. We employed board-certified internal medicine physicians who adjudicated the verbal and demonstrated knowledge items. The adjudicators had no contact with research participants, clinical trial coordinators, intervention nurses, or clinical site nurses. Two adjudicators who were blind to intervention allocation independently scored each patient response as correct or incorrect when compared to the prescription label on the medication container. The initial scores by each adjudicator were compared and revealed moderate to very good agreement. For example, Cohen's Kappa was 0.87 for two adjudicators who scored patient responses to the question, “How many pills of this medicine do you take each day in total?” The Kappa was 0.43 for two adjudicators who scored responses to the question, “How many pills of this medicine do you take each time?” The other verbal and demonstrated knowledge questions had Kappa values between 0.54 and 0.95. When initial adjudications were discordant, the adjudicators met and they successfully resolved all discrepancies.

### 2.6. Measurement of Adherence

A secondary prespecified outcome was adherence. We assessed patient-reported adherence with the Patient Medication Adherence Questionnaire (PMAQ) [37]. Clinical trial coordinators recorded adherence at baseline and then three and six months after randomized allocation. For each daily prescribed medication, clinical trial coordinators asked patients if they missed taking a dose yesterday, the day before yesterday, 3 days ago, or over the past weekend. Participants were scored as being adherent to the medication if they answered “no” to all of the four questions. For purposes of analysis, we constructed a regimen adherence score for each patient: the total number of medicines for which the patient was adherent divided by the total number of medications in the patient's regimen.

### 2.7. Measurement of Satisfaction

Satisfaction with information about medicines was another secondary, prespecified, patient-reported outcome. Clinical trial coordinators asked patients in both intervention groups to rate satisfaction with the information received from the doctor or nurse about medicines during the visits immediately after intervention, at month 3, and at month 6. The response options were “too much,” “about right,” “too little,” “none received,” or “none needed.” The five satisfaction items were a subset of the Satisfaction with Information about Medicines Scales (SIMS) [38]: “what your medicine is called,” “what your medicine is for,” “how to use your medicine,” “whether the medicine has any unwanted effects (side effects),” and “whether the medicine interferes with other medicines.” Responses of “about right” or “none needed” were interpreted as satisfaction. We scored dissatisfaction if the patient reported “too much,” “too little,” or “none received” or if the value was missing.

### 2.8. Measurement of Glycemic Control

HbA1c, a common measure for glycemic control, was another secondary, prespecified outcome. HbA1c was collected from a glycosylated hemoglobin blood test. The blood tests were analyzed at certified clinical laboratories from patient samples drawn at baseline and then during subsequent visit windows that were 3, 6, 9, and 12 months after random allocation. Clinical trial coordinators abstracted HbA1c results from the patient record.

### 2.9. Sample Size

The sample size estimate for the clinical trial was made with the following assumptions. We assumed 45% of patients in the usual care arm would have correct knowledge of their multidrug regimens at six months. The anticipated retention rate at six months was 80%. There were no planned interim analyses. We needed to recruit a sufficient number of patients to have 600 evaluable participants at six months. Under these assumptions, the sample size of 600 (300 per arm) at six months had 82% power to detect a difference of 12% between study arms with a 5% type I error rate.

### 2.10. Randomization Scheme

Research personnel at the clinical trial coordination center generated the random allocation sequence with computer-generated random numbers. The allocation ratio was 1 : 1 with stratification by site, Chicago versus Peoria, and random permuted blocks within site. The coordination center personnel in Champaign, Illinois, transferred the allocation sequence to sequentially numbered, opaque envelopes and then distributed the sealed envelopes to the clinical sites in Chicago and Peoria. Clinical trial coordinators in Chicago and Peoria performed telephone interviews to screen potential participants, confirm eligibility, and obtain verbal consent. Next, the clinical trial coordinators obtained the concealed allocation to Medtable or usual care by opening the sealed envelope. After random allocation, the participant, the clinical trial coordinator, and the clinic personnel were not blind to the study intervention.

### 2.11. Measurement of Covariates

Health literacy was measured by the Rapid Estimate of Adult Literacy in Medicine (REALM), a health word recognition test that involves pronouncing 66 medical terms [39]. Performance on REALM is associated with patient age, medication adherence, and health outcomes [6, 40]. A patient with limited health literacy was defined as having a REALM score of less than 61. We measured fluid mental ability (speed of mental processing) with the Letter and Pattern Comparison tests. Fluid mental ability is vulnerable to aging and is associated with differences in health literacy [41, 42]. We measured patient knowledge about diabetes mellitus with the 24-item Diabetes Knowledge Questionnaire [43]. To adjust for patient self-activation, we assessed the Summary of Diabetes Self-Care Activities (SDSCA) [44]. We assessed illness experience in years when we asked the question, “How long have you had diabetes?” To adjust for health status, we measured comorbidity with the Charlson method and general health status via Short Form-36 [45, 46]. We measured the Medication Regimen Complexity Index (MRCI), a 65-item tool with three domains: medication dosage form, dosing frequency, and additional medication directions [47]. The variables for patient age, gender, race, education, employment, and income were measured by a modified version of the Older Americans Resources and Services (OARS) instrument [48].

### 2.12. Analysis Plan

We analyzed all outcome measures under the principle of intention to treat. To address missing glycosylated hemoglobin (HbA1c) scores, we used the last observation carried forward [49]. Missing satisfaction values were replaced with dissatisfaction values. All other missing outcome measures and missing baseline covariates were replaced using the method of maximum likelihood estimation. Generalized Estimating Equations were used for correlated response data when testing the intervention effects over time with logit link and identity link functions for binary outcomes and continuous outcomes, respectively. When examining the intervention effects within each time visit, we used logistic regression or linear regression models for binary and continuous responses, respectively. All analyses were performed with SAS 9.4 (SAS Institute Inc., Cary, NC). Two-tailed *p* values were calculated for all tests and *p* < 0.05 was the threshold for significance.

The primary analyses evaluated whether the Medtable intervention improved patient outcomes relative to the usual care group. Generalized Estimating Equations included group (Medtable versus usual care), time, group × time, and appropriate covariates. The group × time interaction term evaluated intervention-related benefits that varied with the amount of time exposure to the Medtable collaborative tool. The assumption was that patients might need time to learn to use the tool to structure medication-taking strategies at home and to communicate with providers during office visits. Research site was included in all analyses.

## 3. Results

**Figure 2:**
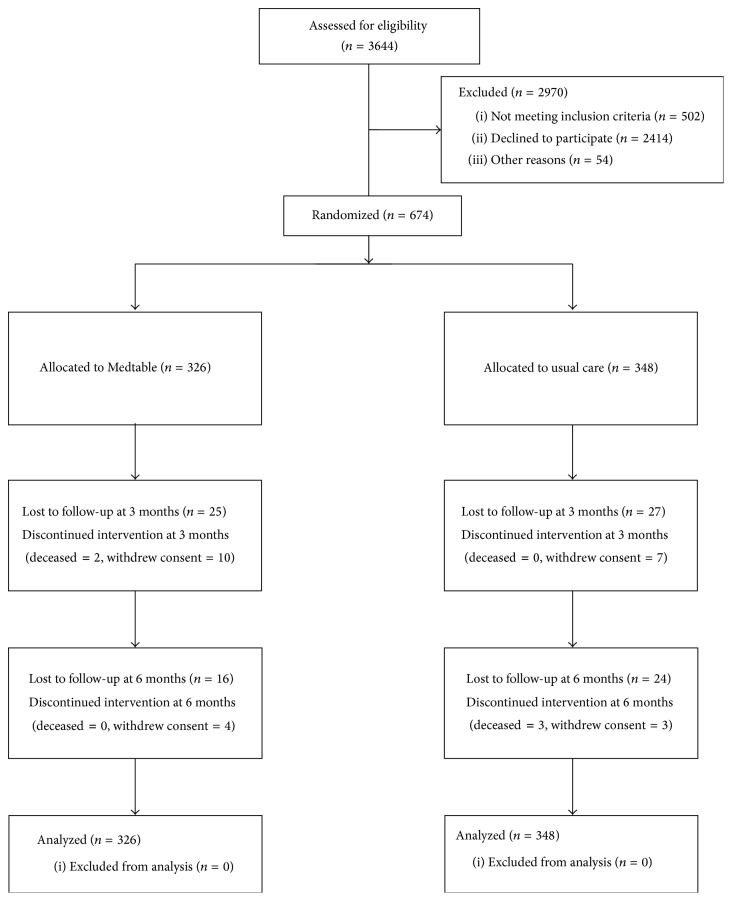
Trial flow diagram.

The researchers performed a clinical trial and screened 3644 outpatients. Clinical trial coordinators recruited participants between September 2011 and October 2013. The trial flow diagram (Figure 2) shows the numbers of patients screened, excluded, randomized, and followed up. The patient-participants who received the randomized intervention, Medtable versus usual care, were comparable at baseline (Table 1) except for years with diabetes mellitus. The characteristics of the participants included age greater than 65 years for 43.3% (292/674), high school or less education for 30.1% (203/674), and limited literacy (as measured by the REALM) for 22.3% (150/674).

One of the primary outcomes of the clinical trial was the effect of the intervention, Medtable versus usual care, on the patients' verbal knowledge of their medication regimen. To score the verbal knowledge, we used adjudicators who were blind to intervention allocation. Adjudicated results in Table 2 are for noninjectable medications. There was no difference between the intervention and control group for “combined verbal knowledge of the regimen for all questions” (Generalized Estimating Equation parameter group, adjusted *p* = 0.3035; parameter group × time, adjusted *p* = 0.6280). Separate analyses for each question within the verbal knowledge score also revealed no consistent effect of the intervention. The only significant effect was for the following: “On a usual day, how many times a day do you take this medicine?” (Generalized Estimating Equation parameter group, adjusted *p* = 0.0373; parameter group × time, adjusted *p* = 0.5294). The analysis of verbal knowledge of the regimen for injectable drugs showed similar results to noninjectable drugs (data available upon request).

The other primary outcome of the trial was the patient's demonstrated knowledge of their medication regimen. The adjudicators who scored the demonstrated knowledge were blind to intervention allocation. The results in Table 3 reveal no difference between the intervention and control group for “combined demonstration knowledge of the regimen for all 4 questions” (Generalized Estimating Equation parameter group, adjusted *p* = 0.3916; parameter group × time, adjusted *p* = 0.8227). Separate analyses for each question within the demonstrated knowledge score exposed no consistent effect of the intervention.

Some evidence for the impact of the intervention on medication knowledge was provided by the measure of medication indication. Adjudicators who were blind to intervention allocation scored the patients' responses to the question, “What is the medicine for?” The results in Table 4 reveal significant increases in correct patient knowledge about indication in the Medtable intervention versus usual care group immediately after the beginning of the intervention that persisted for 6 months (Generalized Estimating Equation parameter group, adjusted p less than 0.0001).

Satisfaction with information about medicines was a prespecified secondary outcome. Patient-reported responses to five satisfaction questions were recorded by research personnel who were not blind to the intervention allocation. The results of the intention-to-treat analysis in Table 5 reveal that patients reported greater satisfaction with Medtable versus usual care at all times. The Generalized Estimating Equation for each satisfaction question included all time points and confirmed the significant increase with Medtable: all adjusted *p* values for group were less than 0.0161.

**Figure 3:**
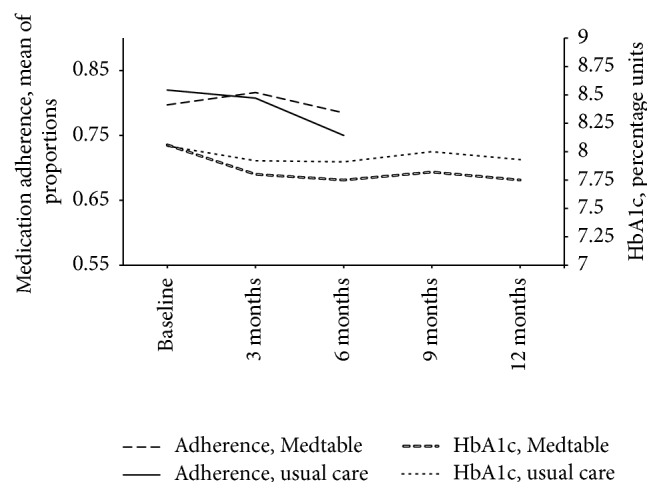
Medication adherence and glycosylated hemoglobin (HbA1c) before and after intervention: Medtable versus usual care.

Medication adherence was a prespecified secondary outcome. Patient-reported adherence was recorded by research personnel who were not blind to the intervention allocation. The results for medication adherence appear in Table 6 and Figure 3. Adherence was greater at baseline in the usual care group and then adherence decreased monotonically over the next 6 months. In contrast, adherence in the Medtable group remained flat and did not deteriorate over time. Figure 3 shows the difference in slopes for the Medtable group and usual care group. The Generalized Estimating Equation for adherence reflects the difference in slopes in the group × time interaction: adjusted *p* = 0.0268. However, the GEE for adherence did not reveal a significant overall effect of Medtable: group adjusted *p* value = 0.7423.

Glycosylated hemoglobin (HbA1c) was a prespecified secondary outcome that was abstracted from the patient record. HbA1c results are in Table 7 and Figure 3. Regardless of the intervention group, patients had significant decreases (improvements) in their HbA1c during their time in the trial: the adjusted parameter estimate for time in the Generalized Estimating Equation had *p* less than 0.0001. There were no significant differences in HbA1c between the intervention groups. In the Generalized Estimating Equation for HbA1c, the parameter for group had adjusted *p* = 0.3639 and the parameter for group × time had adjusted *p* = 0.6079.

Table 8 has knowledge and adherence outcomes within strata defined by limited or adequate literacy. For verbal and demonstrated knowledge of the regimen, there was no apparent effect of Medtable in either stratum. Patients' knowledge of drug indication improved with Medtable regardless of their literacy status. For regimen adherence, the improvements caused by Medtable were seen in patients with adequate literacy and were only demonstrable at the sixth month.

## 4. Discussion

The Medtable intervention increased patient satisfaction with communication about medications during clinic visits. However, there was only mixed evidence that the intervention also improved patients' knowledge about their medications. Knowledge about medication indication improved in the Medtable group. In contrast, the Medtable did not improve verbal or demonstration measures of knowledge about directions for use. The intervention also sustained adherence to medications during the trial while adherence declined in the control group, but the overall difference with the usual care control group was not significant. Finally, the intervention did not influence HbA1c levels, which declined (better glycemic control) equally for the two groups during the trial.

The study results are partially consistent with the process-knowledge model of health literacy [8]. According to this model, improving health knowledge (medication knowledge in our study) should improve self-care behaviors (adherence in this case), which in turn should influence outcomes such as HbA1c. While use of the Medtable by nurses and patients in the clinics influenced collaboration (reflected by improved patient satisfaction) and improved some aspects of patients' medication knowledge (knowledge about indication but not directions for use), the intervention had only limited impact on adherence. Moreover, the intervention did not improve health outcomes as measured by HbA1c. Our results are similar to a recent trial in which a computer-based decision aid designed to support self-care planning among patients with diabetes improved patient perceptions of information clarity and helpfulness, but not health knowledge or outcomes [31].

The finding that the Medtable intervention improved patient satisfaction with provider communication might be important because patient satisfaction is linked to quality and reimbursement [50]. The Medicare Shared Savings Program and other Pay-for-Performance Programs rely on patient satisfaction measures, specifically the Clinician and Group Survey, Consumer Assessment of Healthcare Providers and Systems (CG-CAHPS) [51]. A limitation of our study is the unknown correlation between Satisfaction with Information about Medicines Scales (SIMS) and broader measures of satisfaction, like CG-CAHPS, which are used for value-based purchasing. Future studies should include measures like CG-CAHPS to assess patient satisfaction with the Medtable.

The intervention may have had an attenuated impact on medication knowledge for several reasons. First, performance on both the verbal and the demonstration measures approached ceiling, perhaps reducing the ability of the measures to detect differences between conditions. Second, taking the results at face value, they suggest that usual care practices related to patient education and medication reconciliation at the research site clinics were effective in supporting patients' knowledge about medication.

The limited impact of the intervention on medication adherence may reflect the fact that adherence was self-reported in this study, which can overestimate adherence [52]. More objective measures of adherence might have been more sensitive to potential intervention effects. The intervention may also have had a limited effect on adherence because of its selective effect on participants' medication knowledge. While it is important for patients to know what medications are used for, it is equally if not more important to know how to take the medication, and both groups of participants in the study demonstrated good knowledge about directions for use. In addition, adherence is a complex behavior that is influenced by many factors in addition to medication knowledge, such as patient self-efficacy and cost of the medication. It is also possible that the intervention improved planning for taking medication when working with providers at the clinics, but patients had difficulty implementing these plans at home due to either cost, health, unmeasured socioeconomic factors, or prioritization.

The intervention did not improve HbA1c, which tended to improve equally in both groups. This may well reflect the limited impact of the intervention on medication knowledge and adherence. Also, like medication adherence, HbA1c is influenced by a range of patient factors [53]. Therefore, an intervention designed to improve knowledge and planning for how to take medication might have a limited impact on this outcome, even if it had had a large impact on knowledge. For example, only four of 15 studies investigating impact of communication interventions on patients with cardiovascular disease showed improved clinical outcomes [54]. It is also possible that 6 months was too short for the intervention to produce detectable effects on health outcomes.

One of the limitations of our trial design was the unmasked intervention. For participants assigned to usual care, their clinic nurses may have changed communication and collaborative planning after observation of colleagues who used the Medtable. This phenomenon is encountered in unmasked trials and is called contamination. We attempted to minimize contamination when we blocked the Medtable display in the electronic medical records of participants assigned to usual care. However, some contamination was inevitable. When contamination occurred, there was bias toward the null (increased type II error).

Another limitation of the study is the generalizability of the results. Only 18% (674/3644) of the patients in the screening population provided consent to participate in our trial. The results of our study are most applicable to ambulatory clinic populations that resemble the characteristics reported in Table 1.

The Medtable tool supported provider/patient collaboration related to medication use, as reflected in patient satisfaction with communication, but had limited impact on patient medication knowledge, adherence, and outcomes. A possible reason for this pattern is that the tool as implemented in this study was designed to support collaboration in the clinic but did not support patients at home when taking their medication. Integrating the tool into smartphones or other patient-centered technologies used at home, especially if integrated with provider information technology (e.g., electronic health record patient portal), may support distributed collaboration between providers and patients at home, so that patients can more easily implement plans and update them as medication regimens change.

## Figures and Tables

**Table 1:** Baseline characteristics of 674 outpatients allocated to Medtable or usual care.

	Medtable *N* = 326	Usual care *N* = 348
Age, years, mean (SD)	63.8 (10.3)	63.5 (10.0)

Gender, *n* (%)		
Female	170 (52.1)	202 (58.1)
Male	156 (47.9)	146 (41.9)

Race, *n* (%)		
White	228 (69.9)	214 (61.9)
Black	79 (24.2)	118 (33.9)
Other	19 (5.8)	16 (4.6)

Education, *n* (%)		
High school or less	89 (27.3)	114 (33.0)
Some college or college graduate	237 (72.7)	231 (67.0)

Annual income, *n* (%)		
Less than $20,000	63 (20.0)	79 (24.4)
$20,000–$50,000	109 (34.6)	114 (35.2)
Greater than $50,000	143 (45.4)	131 (40.4)

Employed status, *n* (%)		
Full-time	82 (25.4)	73 (21.1)
Part-time	31 (9.6)	44 (12.7)
Not employed	210 (65.0)	229 (66.2)

REALM, mean (SD)	61.1 (8.8)	61.2 (8.9)

Health literacy, *n* (%)		
Limited, REALM less than 61	77 (23.6)	73 (21.0)
Adequate, REALM 61 and above	249 (76.4)	275 (79.0)

Pattern Comparison test, mean (SD)	27.2 (7.1)	26.5 (7.0)

Letter Comparison test, mean (SD)	17.4 (5.1)	16.9 (4.8)

Years with diabetes mellitus, mean (SD)	13.4 (9.7)	11.7 (9.2)

Diabetes mellitus knowledge, mean (SD)	18.8 (2.9)	18.6 (2.9)

Diabetes self-care activities		
Diet, mean (SD)	4.1 (2.1)	4.3 (2.1)
Exercise, mean (SD)	2.5 (2.1)	2.5 (2.0)
Glucose testing, mean (SD)	4.6 (2.7)	4.6 (2.7)

Comorbidity Index, mean (SD)	2.2 (1.8)	2.3 (1.7)

General health from SF-36, mean (SD)	51.3 (21.1)	50.4 (22.4)

Number of medications per patient, mean (SD)	7.3 (2.8)	7.2 (2.9)

Medication Regimen Complexity Index, mean (SD)	17.7 (7.6)	17.2 (7.7)
Dosage forms, mean (SD)	3.3 (2.5)	3.2 (2.8)
Dosage frequency, mean (SD)	10.9 (4.9)	10.7 (4.7)
Additional directions, mean (SD)	3.5 (2.3)	3.3 (2.4)

REALM: Rapid Estimate of Adult Literacy in Medicine.

**Table 2:** Patient-reported verbal knowledge of the noninjectable medication regimen before and after intervention: Medtable versus usual care.

	Time	Medtable	Usual care	Unadjusted intervention effect (ITT)		Adjusted intervention effect (ITT)	
		Mean (SD)	Mean (SD)	OR (95% CI)	*p* value	OR (95% CI)	*p* value
Combined verbal knowledge of the regimen for all questions	Baseline, preintervention	0.808 (0.175)	0.802 (0.180)	1.03 (0.89, 1.18)	0.7059	1.05 (0.91, 1.21)	0.5470
	Immediate, postintervention	0.812 (0.172)	0.80 (0.176)	1.08 (0.94, 1.25)	0.2598	1.10 (0.96, 1.27)	0.1819
	Month 3	0.808 (0.180)	0.801 (0.168)	1.07 (0.93, 1.23)	0.3641	1.06 (0.92, 1.23)	0.4153
	Month 6	0.806 (0.184)	0.795 (0.179)	1.10 (0.95, 1.26)	0.1968	1.09 (0.94, 1.26)	0.2487

“On a usual day, how many times a day do you take this medicine?”	Baseline, preintervention	0.916 (0.113)	0.906 (0.131)	1.09 (0.89, 1.32)	0.4104	1.09 (0.89, 1.33)	0.4092
	Immediate, postintervention	0.923 (0.104)	0.910 (0.129)	1.12 (0.92, 1.37)	0.2556	1.13 (0.92, 1.38)	0.2576
	Month 3	0.924 (0.113)	0.904 (0.125)	1.31 (1.07, 1.60)	0.0092	1.29 (1.05, 1.59)	0.0142
	Month 6	0.925 (0.103)	0.912 (0.119)	1.26 (1.03, 1.54)	0.0273	1.23 (1.00, 1.52)	0.0492

“How many pills of this medicine do you take each time?”	Baseline, preintervention	0.877 (0.150)	0.881 (0.145)	1.01 (0.85, 1.20)	0.9125	1.04 (0.87, 1.23)	0.6930
	Immediate, postintervention	0.882 (0.147)	0.880 (0.143)	1.06 (0.89, 1.25)	0.5312	1.09 (0.92, 1.30)	0.3175
	Month 3	0.869 (0.161)	0.875 (0.140)	0.99 (0.83, 1.17)	0.8694	1.00 (0.84, 1.19)	0.9826
	Month 6	0.875 (0.153)	0.868 (0.142)	1.05 (0.88, 1.24)	0.5996	1.06 (0.90, 1.26)	0.4807

“How many pills of this medicine do you take each day in total?”	Baseline, preintervention	0.862 (0.147)	0.859 (0.154)	1.01 (0.86, 1.19)	0.8924	1.02 (0.87, 1.20)	0.7857
	Immediate, postintervention	0.866 (0.148)	0.852 (0.152)	1.10 (0.94, 1.29)	0.2376	1.12 (0.95, 1.32)	0.1810
	Month 3	0.867 (0.145)	0.858 (0.146)	1.09 (0.92, 1.28)	0.3222	1.08 (0.92, 1.28)	0.3435
	Month 6	0.870 (0.147)	0.862 (0.152)	1.14 (0.97, 1.34)	0.1201	1.12 (0.95, 1.33)	0.1874

ITT: intention-to-treat analysis. Covariates used for adjustment were health literacy (REALM), Letter Comparison test, Pattern Comparison test, diabetes mellitus knowledge, diabetes self-care: diet, diabetes self-care: exercise, diabetes self-care: glucose testing, years with diabetes mellitus, Comorbidity Index, general health, age, gender, race, patient education, research site, and Medication Regimen Complexity Index.

**Table 3:** Patient-demonstrated knowledge of the medication regimen before and after intervention: Medtable versus usual care.

	Time	Medtable	Usual care	Unadjusted intervention effect (ITT)		Adjusted intervention effect (ITT)	
		Mean (SD)	Mean (SD)	OR (95% CI)	*p* value	OR (95% CI)	*p* value
Combined demonstration knowledge of the regimen for all 4 questions	Baseline, preintervention	0.86 (0.15)	0.85 (0.16)	1.03 (0.88, 1.20)	0.7320	1.03 (0.88, 1.21)	0.7402
	Immediate, postintervention	0.86 (0.15)	0.85 (0.15)	1.08 (0.92, 1.26)	0.3560	1.09 (0.93, 1.29)	0.2799
	Month 3	0.86 (0.15)	0.85 (0.15)	1.07 (0.91, 1.26)	0.3916	1.06 (0.90, 1.25)	0.4814
	Month 6	0.86 (0.14)	0.86 (0.14)	1.08 (0.92, 1.27)	0.3676	1.06 (0.90, 1.26)	0.4763

When compared to the label, the patient demonstrates correct number of pills per dose	Baseline, preintervention	0.895 (0.131)	0.90 (0.13)	0.97 (0.81, 1.17)	0.7728	0.99 (0.82, 1.19)	0.8971
	Immediate, postintervention	0.9 (0.131)	0.90 (0.13)	1.05 (0.87, 1.26)	0.6274	1.09 (0.90, 1.31)	0.3880
	Month 3	0.90 (0.134)	0.89 (0.13)	0.95 (0.79, 1.14)	0.5549	0.95 (0.79, 1.14)	0.5925
	Month 6	0.902 (0.128)	0.91 (0.119)	0.99 (0.82, 1.20)	0.9553	1.00 (0.83, 1.22)	0.9839

When compared to the label, the patient demonstrates correct number of doses per day	Baseline, preintervention	0.89 (0.13)	0.88 (0.14)	1.03 (0.87, 1.23)	0.7262	1.03 (0.86, 1.24)	0.7093
	Immediate, postintervention	0.89 (0.126)	0.88 (0.14)	1.10 (0.92, 1.32)	0.2823	1.12 (0.93, 1.34)	0.2448
	Month 3	0.90 (0.129)	0.88 (0.139)	1.19 (0.99, 1.42)	0.0622	1.18 (0.98, 1.42)	0.0758
	Month 6	0.901 (0.127)	0.90 (0.126)	1.12 (0.93, 1.35)	0.2243	1.10 (0.91, 1.33)	0.3228

The patient demonstrates correct number of pills each day in total	Baseline, preintervention	0.87 (0.143)	0.87 (0.147)	1.04 (0.88, 1.23)	0.6258	1.04 (0.88, 1.23)	0.6504
	Immediate, postintervention	0.878 (0.140)	0.87 (0.14)	1.06 (0.90, 1.25)	0.5046	1.07 (0.90, 1.27)	0.4345
	Month 3	0.875 (0.136)	0.87 (0.14)	1.07 (0.91, 1.27)	0.4020	1.06 (0.90, 1.26)	0.4883
	Month 6	0.880 (0.134)	0.88 (0.135)	1.07 (0.90, 1.26)	0.4623	1.05 (0.88, 1.25)	0.5656

The patient demonstrates correct amount of time (spacing) between doses	Baseline, preintervention	0.89 (0.130)	0.88 (0.147)	1.02 (0.86, 1.21)	0.8340	1.02 (0.85, 1.22)	0.8082
	Immediate, postintervention	0.90 (0.126)	0.88 (0.14)	1.09 (0.91, 1.30)	0.3301	1.11 (0.93, 1.33)	0.2611
	Month 3	0.90 (0.130)	0.88 (0.14)	1.17 (0.98, 1.40)	0.0855	1.17 (0.97, 1.40)	0.1104
	Month 6	0.9 (0.127)	0.89 (0.126)	1.12 (0.93, 1.34)	0.2457	1.09 (0.91, 1.32)	0.3525

ITT: intention-to-treat analysis. Covariates used for adjustment were health literacy (REALM), Letter Comparison test, Pattern Comparison test, diabetes mellitus knowledge, diabetes self-care: diet, diabetes self-care: exercise, diabetes self-care: glucose testing, years with diabetes mellitus, Comorbidity Index, general health, age, gender, race, patient education, research site, and Medication Regimen Complexity Index.

**Table 4:** Patients-reported knowledge about the indications for medicines in their regimen before and after intervention: Medtable versus usual care.

	Time	Medtable	Usual care	Unadjusted intervention effect (ITT)		Adjusted intervention effect (ITT)	
		Mean (SD)	Mean (SD)	OR (95% CI)	*p* value	OR (95% CI)	*p* value
Combined knowledge of the indication for drugs in the regimen, “what is the medicine for?”	Baseline, preintervention	0.87 (0.21)	0.87 (0.20)	1.04 (0.88, 1.23)	0.6815	1.06 (0.89, 1.27)	0.4977
	Immediate, postintervention	0.94 (0.12)	0.88 (0.19)	2.22 (1.80, 2.74)	<0.0001	2.32 (1.86, 2.88)	<0.0001
	Month 3	0.95 (0.12)	0.88 (0.19)	2.34 (1.88, 2.91)	<0.0001	2.45 (1.95, 3.09)	<0.0001
	Month 6	0.96 (0.09)	0.91 (0.17)	2.35 (1.86, 2.98)	<0.0001	2.53 (1.97, 3.25)	<0.0001

ITT: intention-to-treat analysis. Covariates used for adjustment were health literacy (REALM), Letter Comparison test, Pattern Comparison test, diabetes mellitus knowledge, diabetes self-care: diet, diabetes self-care: exercise, diabetes self-care: glucose testing, years with diabetes mellitus, Comorbidity Index, general health, age, gender, race, patient education, research site, and Medication Regimen Complexity Index.

**Table 5:** Patient-reported satisfaction with information about medicines after intervention: Medtable versus usual care.

Satisfaction question	Time	Medtable *N* = 326	Usual care *N* = 348	Unadjusted intervention effect (ITT)		Adjusted intervention effect (ITT)	
		*n* (%)	*n* (%)	OR (95% CI)	*p* value	OR (95% CI)	*p* value
“What your medicine is called”	Immediate, postintervention	314 (96.3%)	289 (83.0%)	5.34 (2.81, 10.14)	<0.0001	5.84 (3.00, 11.38)	<0.0001
	Month 3	281 (86.2%)	246 (70.7%)	2.60 (1.75, 3.83)	<0.0001	2.85 (1.88, 4.31)	<0.0001
	Month 6	253 (77.6%)	237 (68.1%)	1.62 (1.15, 2.29)	0.0058	1.57 (1.09, 2.25)	0.0146

“What your medicine is for”	Immediate, postintervention	319 (97.9%)	298 (85.6%)	7.65 (3.41, 17.13)	<0.0001	8.91 (3.87, 20.53)	0.0001
	Month 3	285 (87.4%)	247 (71.0%)	2.84 (1.90, 4.24)	<0.0001	3.10 (2.02, 4.74)	<0.0001
	Month 6	261 (80.1%)	238 (68.4%)	1.86 (1.30, 2.64)	0.0006	1.82 (1.26, 2.63)	0.0014

“How to use your medicine”	Immediate, postintervention	315 (96.6%)	279 (80.2%)	7.08 (3.67, 13.65)	<0.0001	8.03 (4.07, 15.85)	<0.0001
	Month 3	285 (87.4%)	233 (67.0%)	3.43 (2.31, 5.10)	<0.0001	3.83 (2.52, 5.84)	<0.0001
	Month 6	254 (77.9%)	225 (64.7%)	1.93 (1.37, 2.71)	0.0002	1.19 (1.32, 2.70)	0.0005

“Whether the medicine has any unwanted effects (side effects)”	Immediate, postintervention	225 (69.0%)	229 (65.8%)	1.16 (0.84, 1.60)	0.3740	1.18 (0.84, 1.66)	0.3495
	Month 3	207 (63.5%)	188 (54.0%)	1.48 (1.09, 2.02)	0.0127	1.61 (1.16, 2.25)	0.0048
	Month 6	194 (59.5%)	190 (54.6%)	1.22 (0.90, 1.70)	0.1983	1.20 (0.87, 1.65)	0.2622

“Whether the medicine interferes with other medicines”	Immediate, postintervention	212 (65.0%)	216 (62.1%)	1.14 (0.83, 1.56)	0.4249	1.10 (0.78, 1.55)	0.5803
	Month 3	189 (58.0%)	171 (49.1%)	1.43 (1.05, 1.94)	0.0217	1.55 (1.11, 2.16)	0.0096
	Month 6	187 (57.4%)	167 (48.0%)	1.46 (1.08, 1.98)	0.0150	1.50 (1.08, 2.08)	0.0147

ITT: intention-to-treat analysis. Covariates used for adjustment were health literacy (REALM), Letter Comparison test, Pattern Comparison test, diabetes mellitus knowledge, diabetes self-care: diet, diabetes self-care: exercise, diabetes self-care: glucose testing, years with diabetes mellitus, Comorbidity Index, general health, age, gender, race, patient education, research site, and Medication Regimen Complexity Index.

**Table 6:** Patients-reported adherence to their medication regimen before and after intervention: Medtable versus usual care.

	Time	Medtable	Usual care	Unadjusted intervention effect (ITT)		Adjusted intervention effect (ITT)	
		Mean (SD)	Mean (SD)	OR (95% CI)	*p* value	OR (95% CI)	*p* value
Regimen adherence score	Baseline, preintervention	0.80 (0.26)	0.84 (0.23)	0.83 (0.72, 0.96)	0.0115	0.78 (0.68, 0.91)	0.001
	Month 3	0.82 (0.19)	0.81 (0.20)	1.09 (0.95, 1.25)	0.2028	1.05 (0.92, 1.21)	0.4636
	Month 6	0.80 (0.20)	0.75 (0.21)	1.17 (1.03, 1.32)	0.0156	1.13 (0.999, 1.29)	0.0526

ITT: intention-to-treat analysis. Covariates used for adjustment were health literacy (REALM), Letter Comparison test, Pattern Comparison test, diabetes mellitus knowledge, diabetes self-care: diet, diabetes self-care: exercise, diabetes self-care: glucose testing, years with diabetes mellitus, Comorbidity Index, general health, age, gender, race, patient education, research site, and Medication Regimen Complexity Index.

**Table 7:** Glycosylated hemoglobin (HbA1c) before and after intervention: Medtable versus usual care.

Time	Medtable	Usual care	Unadjusted intervention effect (ITT)		Adjusted intervention effect (ITT)	
	Mean (SD)	Mean (SD)	Coefficient (95% CI)	*p* value	Coefficient (95% CI)	*p* value
Baseline, preintervention	8.06 (1.55)	8.05 (1.63)	0.01 (−0.23, 0.25)	0.9506	0.04 (−0.19, 0.27)	0.7164
Month 3	7.80 (1.37)	7.92 (1.61)	−0.09 (−0.33, 0.14)	0.4508	−0.09 (−0.32, 0.13)	0.4090
Month 6	7.75 (1.41)	7.91 (1.61)	−0.14 (−0.37, 0.10)	0.2535	−0.15 (−0.38, 0.07)	0.1823
Month 9	7.82 (1.46)	8.0 (1.69)	−0.14 (−0.40, 0.11)	0.2680	−0.16 (−0.39, 0.07)	0.1811
Month 12	7.75 (1.33)	7.93 (1.64)	−0.12 (−0.35, 0.11)	0.3130	−0.15 (−0.37, 0.07)	0.1904

ITT: intention-to-treat analysis. Covariates used for adjustment were health literacy (REALM), Letter Comparison test, Pattern Comparison test, diabetes mellitus knowledge, diabetes self-care: diet, diabetes self-care: exercise, diabetes self-care: glucose testing, years with diabetes mellitus, Comorbidity Index, general health, age, gender, race, patient education, research site, and Medication Regimen Complexity Index.

**Table 8:** Stratified analysis by patients' literacy status for knowledge of and adherence to the noninjectable medication regimen before and after intervention: Medtable versus usual care.

	Time	Health literacy	Medtable	Usual care	Unadjusted intervention effect (ITT)	
			Mean (SD)	Mean (SD)	OR (95% CI)	*p* value
Combined verbal knowledge of the regimen for all questions	Baseline, preintervention	Limited	0.81 (0.17)	0.82 (0.17)	0.89 (0.66, 1.20)	0.4457
		Adequate	0.81 (0.18)	0.80 (0.18)	1.07 (0.91, 1.25)	0.4332
	Immediate, postintervention	Limited	0.82 (0.15)	0.82 (0.16)	0.92 (0.68, 1.24)	0.5775
		Adequate	0.81 (0.18)	0.79 (0.18)	1.13 (0.96, 1.32)	0.1341
	Month 3	Limited	0.82 (0.16)	0.81 (0.17)	1.06 (0.78, 1.44)	0.6997
		Adequate	0.80 (0.19)	0.80 (0.17)	1.06 (0.90, 1.25)	0.4644
	Month 6	Limited	0.80 (0.17)	0.83 (0.19)	0.94 (0.69, 1.28)	0.6845
		Adequate	0.81 (0.19)	0.79 (0.18)	1.14 (0.97, 1.34)	0.1061

Combined demonstration knowledge of the regimen for all 4 questions	Baseline, preintervention	Limited	0.86 (0.15)	0.87 (0.16)	0.83 (0.59, 1.17)	0.2828
		Adequate	0.85 (0.15)	0.84 (0.16)	1.08 (0.91, 1.29)	0.3790
	Immediate, postintervention	Limited	0.87 (0.14)	0.88 (0.14)	0.77 (0.54, 1.09)	0.1399
		Adequate	0.86 (0.15)	0.84 (0.16)	1.17 (0.98, 1.40)	0.0838
	Month 3	Limited	0.86 (0.15)	0.86 (0.16)	1.01 (0.72, 1.43)	0.9444
		Adequate	0.86 (0.15)	0.85 (0.14)	1.09 (0.91, 1.30)	0.3630
	Month 6	Limited	0.86 (0.15)	0.89 (0.13)	0.88 (0.62, 1.25)	0.4740
		Adequate	0.87 (0.14)	0.86 (0.14)	1.14 (0.95, 1.37)	0.1711

Combined knowledge of the indication for drugs in the regimen, “what is the medicine for?”	Baseline, preintervention	Limited	0.83 (0.26)	0.83 (0.24)	1.09 (0.79, 1.50)	0.6025
		Adequate	0.89 (0.19)	0.88 (0.19)	1.04 (0.85, 1.27)	0.6921
	Immediate, postintervention	Limited	0.93 (0.14)	0.84 (0.23)	2.44 (1.66, 3.59)	<0.0001
		Adequate	0.95 (0.12)	0.89 (0.18)	2.20 (1.72, 2.82)	<0.0001
	Month 3	Limited	0.94 (0.13)	0.83 (0.24)	2.56 (1.73, 3.80)	<0.0001
		Adequate	0.96 (0.11)	0.90 (0.17)	2.33 (1.79, 3.04)	<0.0001
	Month 6	Limited	0.94 (0.11)	0.85 (0.23)	2.29 (1.53, 3.44)	<0.0001
		Adequate	0.97 (0.09)	0.92 (0.16)	2.51 (1.87, 3.37)	<0.0001

Regimen adherence score	Baseline, preintervention	Limited	0.76 (0.28)	0.80 (0.24)	0.68 (0.51, 0.90)	0.0077
		Adequate	0.81 (0.25)	0.83 (0.22)	0.91 (0.77, 1.07)	0.2438
	Month 3	Limited	0.80 (0.21)	0.80 (0.19)	0.97 (0.74, 1.27)	0.8269
		Adequate	0.83 (0.19)	0.81 (0.20)	1.15 (0.98, 1.34)	0.0858
	Month 6	Limited	0.78 (0.22)	0.76 (0.18)	0.99 (0.77, 1.28)	0.9656
		Adequate	0.81 (0.19)	0.75 (0.21)	1.23 (1.07, 1.42)	0.0040

REALM: Rapid Estimate of Adult Literacy in Medicine. Patients with limited literacy had REALM scores less than 61. Adequate literacy was a REALM score of 61 and above. ITT: intention-to-treat analysis.

## References

[1] American Diabetes Association (2009). Standards of medical care in diabetes—2009. *Diabetes Care*.

[2] Aspden P., Wolcott J., Bootman J. L., Croenwett L. R. (2007). *Preventing Medication Errors*.

[3] Osterberg L., Blaschke T. (2005). Adherence to medication. *The New England Journal of Medicine*.

[4] Paasche-Orlow M. K., Schillinger D., Greene S. M., Wagner E. H. (2006). How health care systems can begin to address the challenge of limited literacy. *Journal of General Internal Medicine*.

[5] Wolf M. S., Curtis L. M., Waite K. (2011). Helping patients simplify and safely use complex prescription regimens. *Archives of Internal Medicine*.

[6] DeWalt D. A., Malone R. M., Bryant M. E. (2006). A heart failure self-management program for patients of all literacy levels: a randomized, controlled trial [ISRCTN11535170]. *BMC Health Services Research*.

[7] Wolf M. S., Wilson E. A. H., Rapp D. N. (2009). Literacy and learning in health care. *Pediatrics*.

[8] Chin J., Morrow D. G., Stine-Morrow E. A. L., Conner-Garcia T., Graumlich J. F., Murray M. D. (2011). The process-knowledge model of health literacy: evidence from a componential analysis of two commonly used measures. *Journal of Health Communication*.

[9] Park D. C., Jones T. R., Fisk A. D., Rogers W. A. (1997). Medication adherence and aging. *Handbook of Human Factors and the Older Adult*.

[10] Bodenheimer T., Lorig K., Holman H., Grumbach K. (2002). Patient self-management of chronic disease in primary care. *The Journal of the American Medical Association*.

[11] Wagner E. H., Bennett S. M., Austin B. T., Greene S. M., Schaefer J. K., Vonkorff M. (2005). Finding common ground: patient-centeredness and evidence-based chronic illness care. *Journal of Alternative and Complementary Medicine*.

[12] Clark H. H. (1996). *Using Language*.

[13] Gerwing J., Indseth T., Gulbrandsen P. (2016). A microanalysis of the clarity of information in physicians' and patients' discussions of treatment plans with and without language barriers. *Patient Education and Counseling*.

[14] Tarn D. M., Heritage J., Paterniti D. A., Hays R. D., Kravitz R. L., Wenger N. S. (2006). Physician communication when prescribing new medications. *Archives of Internal Medicine*.

[15] Stewart M. A. (1995). Effective physician-patient communication and health outcomes: a review. *Canadian Medical Association Journal*.

[16] Schillinger D., Piette J., Grumbach K. (2003). Closing the loop: physician communication with diabetic patients who have low health literacy. *Archives of Internal Medicine*.

[17] Tarn D. M., Paterniti D. A., Kravitz R. L., Fein S., Wenger N. S. (2009). How do physicians conduct medication reviews?. *Journal of General Internal Medicine*.

[18] Skinner T. C., Barnard K., Cradock S., Parkin T. (2007). Patient and professional accuracy of recalled treatment decisions in out-patient consultations. *Diabetic Medicine*.

[19] Schillinger D., Wang F., Rodriguez M., Bindman A., Machtinger E. L. (2006). The importance of establishing regimen concordance in preventing medication errors in anticoagulant care. *Journal of Health Communication*.

[20] Heisler M., Vijan S., Anderson R. M., Ubel P. A., Bernstein S. J., Hofer T. P. (2003). When do patients and their physicians agree on diabetes treatment goals and strategies, and what difference does it make?. *Journal of General Internal Medicine*.

[21] Schillinger D., Grumbach K., Piette J. (2002). Association of health literacy with diabetes outcomes. *The Journal of the American Medical Association*.

[22] Persell S. D., Osborn C. Y., Richard R., Skripkauskas S., Wolf M. S. (2007). Limited health literacy is a barrier to medication reconciliation in ambulatory care. *Journal of General Internal Medicine*.

[23] Johnson A., Sandford J., Tyndall J. (2003). Written and verbal information versus verbal information only for patients being discharged from acute hospital settings to home. *Cochrane Database of Systematic Reviews*.

[24] Kripalani S., Robertson R., Love-Ghaffari M. H. (2007). Development of an illustrated medication schedule as a low-literacy patient education tool. *Patient Education and Counseling*.

[25] Cordasco K. M., Asch S. M., Bell D. S. (2009). A low-literacy medication education tool for safety-net hospital patients. *American Journal of Preventive Medicine*.

[26] Machtinger E. L., Wang F., Chen L.-L., Rodriguez M., Wu S., Schillinger D. (2007). A visual medication schedule to improve anticoagulation control: a randomized, controlled trial. *The Joint Commission Journal on Quality and Patient Safety*.

[27] Morrow D. G., Conner-Garcia T., Graumlich J. F. (2012). An EMR-based tool to support collaborative planning for medication use among adults with diabetes: design of a multi-site randomized control trial. *Contemporary Clinical Trials*.

[28] Hazlehurst B., Gorman P. N., McMullen C. K. (2008). Distributed cognition: an alternative model of cognition for medical informatics. *International Journal of Medical Informatics*.

[29] Sapkota S., Brien J.-A. E., Greenfield J. R., Aslani P. (2015). A systematic review of interventions addressing adherence to anti-diabetic medications in patients with type 2 diabetes—components of interventions. *PLoS ONE*.

[30] Varming A. R., Hansen U. M., Andrésdóttir G., Husted G. R., Willaing I. (2015). Empowerment, motivation, and medical adherence (EMMA): the feasibility of a program for patient-centered consultations to support medication adherence and blood glucose control in adults with type 2 diabetes. *Patient Preference and Adherence*.

[31] Heisler M., Choi H., Palmisano G. (2014). Comparison of community health worker-led diabetes medication decision-making support for low-income latino and african american adults with diabetes using E-health tools versus print materials. *Annals of Internal Medicine*.

[32] Farmer A., Hardeman W., Hughes D. (2012). An explanatory randomised controlled trial of a nurse-led, consultation-based intervention to support patients with adherence to taking glucose lowering medication for type 2 diabetes. *BMC Family Practice*.

[33] O'Connor P. J., Schmittdiel J. A., Pathak R. D. (2014). Randomized trial of telephone outreach to improve medication adherence and metabolic control in adults with diabetes. *Diabetes Care*.

[34] Callahan C. M., Unverzagt F. W., Hui S. L., Perkins A. J., Hendrie H. C. (2002). Six-item screener to identify cognitive impairment among potential subjects for clinical research. *Medical Care*.

[35] Davis T. C., Federman A. D., Bass P. F. (2009). Improving patient understanding of prescription drug label instructions. *Journal of General Internal Medicine*.

[36] Persell S. D., Heiman H. L., Weingart S. N. (2004). Understanding of drug indications by ambulatory care patients. *American Journal of Health-System Pharmacy*.

[37] Wolf M. S., Davis T. C., Osborn C. Y., Skripkauskas S., Bennett C. L., Makoul G. (2007). Literacy, self-efficacy, and HIV medication adherence. *Patient Education and Counseling*.

[38] Horne R., Hankins M., Jenkins R. (2001). The Satisfaction with Information about Medicines Scale (SIMS): a new measurement tool for audit and research. *Quality in Health Care*.

[39] Davis T. C., Long S. W., Jackson R. H. (1993). Rapid estimate of adult literacy in medicine: a shortened screening instrument. *Family Medicine*.

[40] Paasche-Orlow M. K., Parker R. M., Gazmararian J. A., Nielsen-Bohlman L. T., Rudd R. R. (2005). The prevalence of limited health literacy. *Journal of General Internal Medicine*.

[41] Levinthal B. R., Morrow D. G., Tu W., Wu J., Murray M. D. (2008). Cognition and health literacy in patients with hypertension. *Journal of General Internal Medicine*.

[42] Salthouse T. A. (1991). Mediation of adult age differences in cognition by reductions in working memory and speed of processing. *Psychological Science*.

[43] Garcia A. A., Villagomez E. T., Brown S. A., Kouzekanani K., Hanis C. L. (2001). The Starr County Diabetes Education Study: development of the Spanish-language diabetes knowledge questionnaire. *Diabetes Care*.

[44] Toobert D. J., Hampson S. E., Glasgow R. E. (2000). The summary of diabetes self-care activities measure: results from 7 studies and a revised scale. *Diabetes Care*.

[45] Charlson M. E., Pompei P., Ales K. L., MacKenzie C. R. (1987). A new method of classifying prognostic comorbidity in longitudinal studies: development and validation. *Journal of Chronic Diseases*.

[46] Ware J. E., Sherbourne C. D. (1992). The MOS 36-item short-form health survey (Sf-36). I. conceptual framework and item selection. *Medical Care*.

[47] George J., Phun Y.-T., Bailey M. J., Kong D. C. M., Stewart K. (2004). Development and validation of the medication regimen complexity index. *The Annals of Pharmacotherapy*.

[48] Fillenbaum G. (1988). *Multidimensional Functional Assessment of Older Adults*.

[49] U.S. Food and Drug Administration—Center for Drug Evaluation and Research (2008). *Guidance for Industry, Diabetes Mellitus: Developing Drugs and Therapeutic Biologics for Treatment and Prevention*.

[50] Press I., Fullam F. (2011). Patient satisfaction in pay for performance programs. *Quality Management in Health Care*.

[51] The Shaller Consulting Group and Robert Wood Johnson Foundation Forces Driving Implementation of the CAHPS Clinician & Group Survey. http://www.rwjf.org/content/dam/farm/reports/issue_briefs/2013/rwjf72668.

[52] Nieuwenhuis M. M., Jaarsma T., van Veldhuisen D. J., van der Wal M. H. (2012). Self-reported versus ‘true’ adherence in heart failure patients: a study using the Medication Event Monitoring System. *Netherlands Heart Journal*.

[53] Balkau B., Calvi-Gries F., Freemantle N., Vincent M., Pilorget V., Home P. D. (2015). Predictors of HbA1c over 4 years in people with type 2 diabetes starting insulin therapies: the CREDIT study. *Diabetes Research and Clinical Practice*.

[54] Schoenthaler A., Kalet A., Nicholson J., Lipkin M. (2014). Does improving patient-practitioner communication improve clinical outcomes in patients with cardiovascular diseases? A systematic review of the evidence. *Patient Education and Counseling*.

